# Cardiac computed tomography assessment of congenital aortic diseases: a case series

**DOI:** 10.1093/ehjcr/ytad155

**Published:** 2023-03-30

**Authors:** Nilda Espinola-Zavaleta, Javier Ivan Armenta-Moreno, Jorge Alberto Silva-Estrada, Javier Serrano-Roman, Adrian Espejel-Guzman, Valente Fernandez-Badillo, Daniel Alejandro Navarro-Martinez, Roberto Cano-Zarate

**Affiliations:** Department of Nuclear Cardiology, National Institute of Cardiology Ignacio Chavez, Juan Badiano Nº 1, Colonia Seccion XVI, Tlalpan, 14080 Mexico City, Mexico; Department of Echocardiography, ABC Medical Center, IAP, Sur 136 116, Colonia Las Americas, Alvaro Obregon, 01120 Mexico City, Mexico; Department of Nuclear Cardiology, National Institute of Cardiology Ignacio Chavez, Juan Badiano Nº 1, Colonia Seccion XVI, Tlalpan, 14080 Mexico City, Mexico; Department of Cardiovascular Imaging, National Institute of Cardiology Ignacio Chavez, Juan Badiano Nº 1, Colonia Seccion XVI, Tlalpan, 14080 Mexico City, Mexico; Department of Nuclear Cardiology, National Institute of Cardiology Ignacio Chavez, Juan Badiano Nº 1, Colonia Seccion XVI, Tlalpan, 14080 Mexico City, Mexico; Department of Nuclear Cardiology, National Institute of Cardiology Ignacio Chavez, Juan Badiano Nº 1, Colonia Seccion XVI, Tlalpan, 14080 Mexico City, Mexico; Department of Nuclear Cardiology, National Institute of Cardiology Ignacio Chavez, Juan Badiano Nº 1, Colonia Seccion XVI, Tlalpan, 14080 Mexico City, Mexico; Department of Nuclear Cardiology, National Institute of Cardiology Ignacio Chavez, Juan Badiano Nº 1, Colonia Seccion XVI, Tlalpan, 14080 Mexico City, Mexico; Department of Magnetic Resonance, National Institute of Cardiology Ignacio Chavez, Juan Badiano Nº 1, Colonia Seccion XVI, Tlalpan, 14080 Mexico City, Mexico

**Keywords:** Congenital aortic diseases, Cardiac computed tomography angiography, Vascular ring, Aortic coarctation, Case series

## Abstract

**Background:**

Congenital aortic diseases (CAoD) encompass a wide variety of disorders that range from asymptomatic findings to life-threatening conditions. Multiple imaging techniques are available for the assessment of CAoD.

**Case summary:**

We present seven case reports of congenital aortic diseases, including obstructions in the aortic arch (coarctation, hypoplasia, and interruption) and vascular rings, in which the clinical manifestations throughout the cases are discussed, highlighting the heterogeneity of the symptoms.

**Discussion:**

Multi-imaging techniques are indispensable for the assessment of CAoD, where cardiac computed tomography angiography is the main modality for rapid acquisition of three-dimensional volume-rendered images for optimal surgical planning.

**Learning points**

- Cardiovascular computed tomography is first-line non-invasive imaging modality in the diagnosis, selection of optimal surgical planning, and follow-up of congenital aortic diseases.
- The choice of imaging modality for an individual patient should be determined by age, diagnosis, and clinical condition.

## Introduction

Congenital aortic diseases (CAoD) encompass a wide variety of disorders that range from asymptomatic findings to life-threatening conditions.^1,2^ Multiple imaging techniques are available for the assessment of CAoD. Transthoracic echocardiography (TTE) is generally used as the first approach in these pathologies, but cardiac computed tomography angiography (CCTA) provides a more adequate visualization of vascular structures with optimal spatial resolution, being the first-line imaging modality.^1,3^ Moreover, rapid acquisition of three-dimensional volume-rendered images are extremely valuable for an optimal surgical planning.

In this case series, we present seven case reports of CAoD, including vascular rings, interrupting arches, aortic coarctation, and hypoplasia, highlighting the fundamental role played by the CCTA in the timely diagnosis and management of these abnormalities.

## Timeline

**Table:** 

Case 1
Day 1. 3-Year-old male presented with inspiratory stridor and dysphagia Day 2. Echocardiogram: right aortic arch. Cardiac computed tomography angiography (CCTA): complete vascular ring, a left Kommerell diverticulum, and persistent ductus arteriosus Day 6. Hybrid repair with surgical ligation of the ligamentum arteriosum and resection of Kommerell diverticulum Day 20. Follow-up, without complications
Case 2
Day 1. 18-Month-old female with shortness of breath, acrocyanosis, diaphoresis Day 2. CCTA: complete loose vascular ring, a right Kommerell diverticulum Day 5. Surgical management, hybrid repair with surgical ligation of the ligamentum arteriosum, and resection of Kommerell diverticulum Month 1. On follow-up, the patient remained haemodynamically stable.
Case 3
Day 1. 4-Day-old infant male with increased work of breathing and generalized pallor Day 3. CCTA: displayed an interrupted aortic arch type A and a patent ductus arteriosus (PDA) with slight stenosis Day 5. Endovascular procedure, the ligamentum arteriosum was double ligated, and the interrupted aortic arch was repaired by the anterior patch augmentation technique. Month 1. The patient was haemodynamically stable.
Case 4
Day 1. 5-Day-old infant was admitted to sudden circulatory shock. Echocardiogram: interrupted aortic arch and a large ventricular septal defect Day 2. CCTA: interrupted aortic arch type B and a right isolated subclavian artery Day 5. Surgical correction with mobilization of the descending aorta and wide anastomosis of the same with the ascending aorta Day 7. Unfortunately, the patient died in the post-operative period.
Case 5
Day 1. 50-Year-old man with a history of resistant hypertension and dyspnoea. Chest X-ray: showed mild cardiomegaly Day 2. Echocardiogram: revealed aortic coarctation Day 3. CCTA: showed a focal severe coarctation with multiple collateral arteries Day 7. Balloon angioplasty with colocation of two stents was performed. Month 6. Follow-up was adequate, requiring only one hypotensive drug.
Case 6
Day 1. 3-Day-old male with a systolic murmur Day 2. Echocardiogram: revealed hypoplastic left ventricle syndrome Day 4. CCTA: displayed the ascending aorta and aortic arch with pre-ductal aortic coarctation, and hypoplastic left ventricle syndrome Day 8. Surgical resection of the coarctation with an end-to-end anastomosis Month 2. Follow-up, clinically stable
Case 7
Day 1. 9-Month-old male with acrocyanosis and inadequate weight gain Day 3. CCTA: a complete vascular ring made up of a right (dominant) and double aortic arches Day 7. Cardiac catheterization, a stent was placed in the left aortopulmonary collateral Month 3. Follow-up was adequate

### Case 1

A 3-year-old male with a history of recurrent pneumonia, inspiratory stridor, and dysphagia. On arrival, the patient presented a blood pressure of 100/60 mmHg, was tachypnoeic (35 breaths/min), and had an oxygen saturation of 87% on room air. Cardiac physical examination revealed regular rate and rhythm, with an opening snap and aortic systolic ejection murmur.

**Figure 1:**
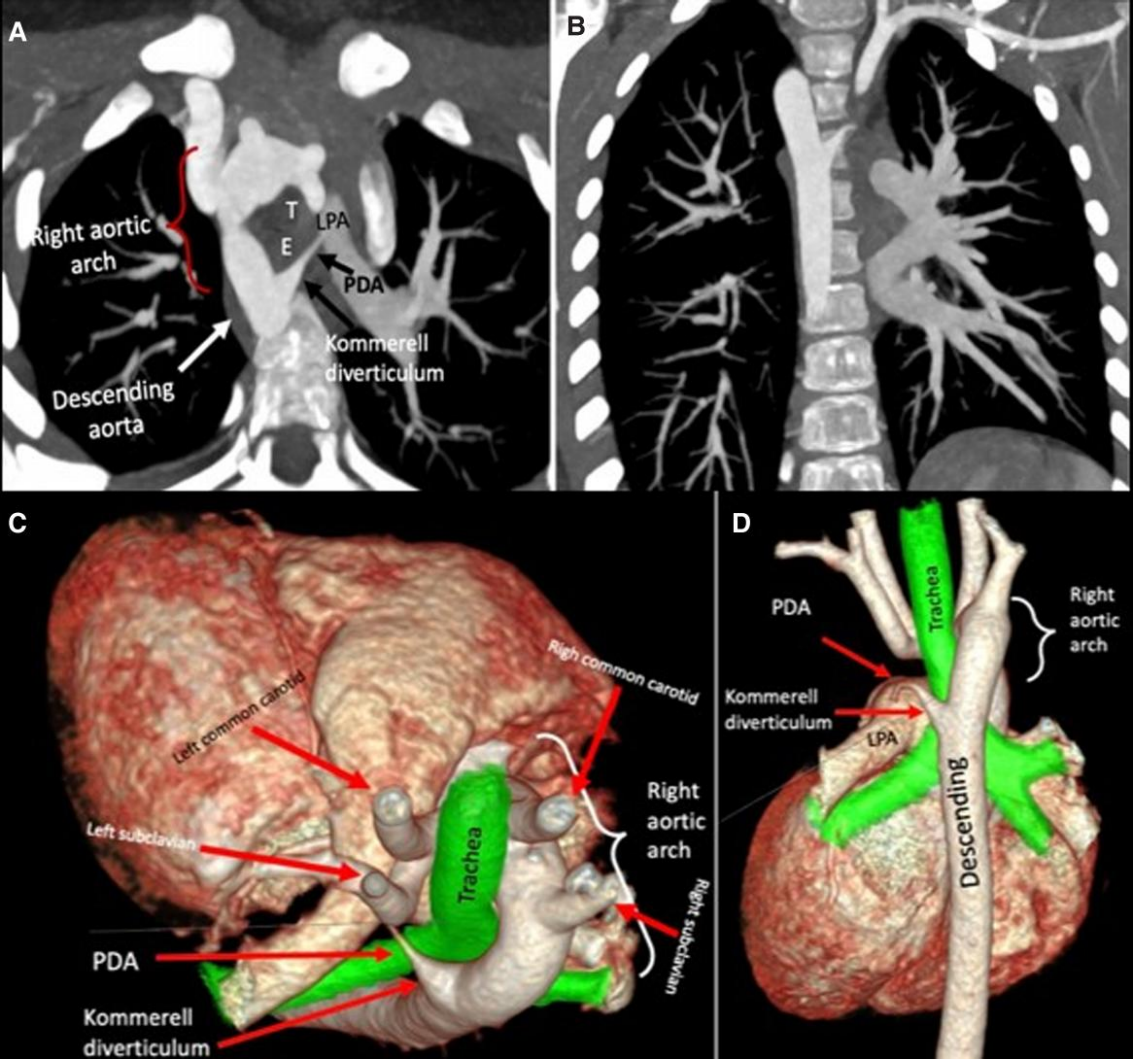
(*A*) The axial oblique reformatted maximum intensity projection image from cardiac computed tomography angiography shows a complete vascular ring made up of a right aortic arch, a left Kommerell diverticulum, and a retroesophageal persistent ductus arteriosus. This anomaly is the result of incomplete regression of the 4th left aortic arch distal to the origin of the left subclavian artery. Abbreviations: LPA, left pulmonary artery; T, trachea; E, oesophagus; PDA, persistent ductus arteriosus. (*B*) Coronal oblique reformatted maximum intensity projection image demonstrates a right ascending aorta with left Kommerell diverticulum. (*C* and *D*) Three-dimensional volume-rendered image in superior and posterior views shows trachea and oesophagus fully surrounded by vascular structures: ascending aorta, right aortic arch, left Kommerell diverticulum, left patent ductus arteriosus, and left pulmonary artery.

Preliminary laboratory work revealed normal values. The initial TTE demonstrated a right-sided aortic arch. A CCTA was then requested to investigate this finding, revealing a complete vascular ring made up of a right aortic arch without obstructions, a left Kommerell diverticulum, interventricular septal aneurysm with a septal defect of 6 mm, and a retroesophageal persistent ductus arteriosus (PDA) (*Figure 1A–D*).

He was admitted for management with diuretics, and posterior hybrid repair with surgical ligation of the ligamentum arteriosum, and resection of Kommerell diverticulum. Follow-up at 3 weeks showed adequate evolution, and the patient remained haemodynamically stable.

### Case 2

**Figure 2:**
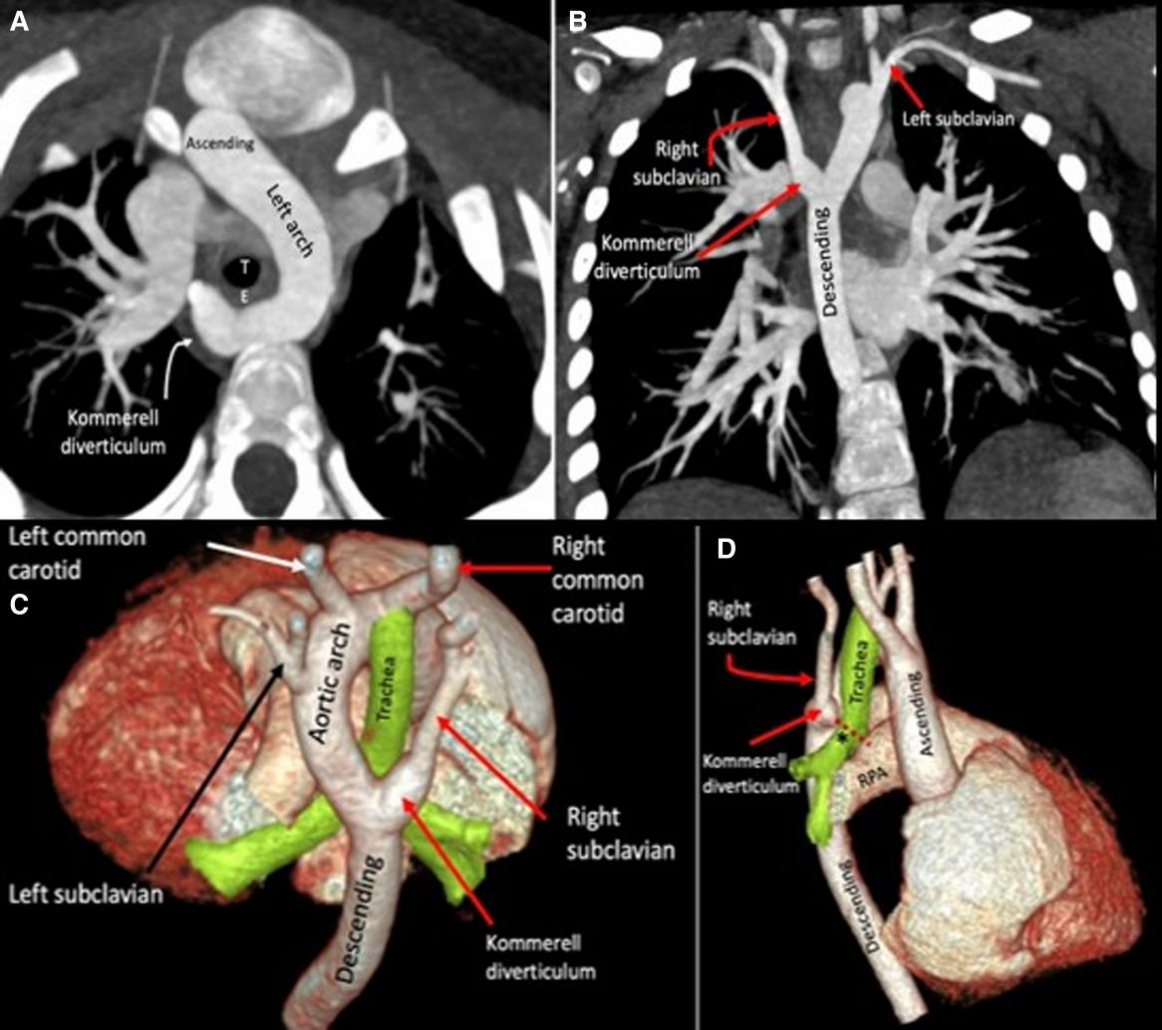
(*A*) The axial oblique reformatted maximum intensity projection image from cardiac computed tomography angiography displays a complete loose vascular ring made up of a left aortic arch, a right Kommerell diverticulum, and a right ligamentum arteriosum as a result of incomplete regression of the 4th right aortic arch between the origin of the right common carotid artery and the right subclavian artery. (*B*) Coronal oblique reformatted maximum intensity projection image CT shows a circumflex arch: a left aortic arch with a right descending aorta. (*C* and *D*) Three-dimensional volume-rendered image in posterosuperior and right lateral views shows trachea and oesophagus fully surrounded by ascending aorta, left aortic arch (circumflex), right Kommerell diverticulum and right ligamentum arteriosum.

An 18-month-old female presented difficulty breathing, acrocyanosis, and diaphoresis during breastfeeding and repeated episodes of respiratory distress over the last 6 months. Cardiac exam revealed regular rate and rhythm, without cardiac murmurs, and pulse oximetry was 93% on room air. An initial echocardiogram ruled out any cardiac malformation. A CCTA was performed and evidenced a complete loose vascular ring made up of a left aortic arch, a right Kommerell diverticulum, and a right ligamentum arteriosum (*Figure 2A–D*).

The surgical team decided on a hybrid repair with surgical ligation of the ligamentum arteriosum, and resection of Kommerell diverticulum. Upon follow-up at 4 weeks, the patient was clinically stable.

### Case 3

A four-day-old infant born at full term after a normal pregnancy began to manifest increased work of breathing, generalized pallor, and a decreased level of consciousness. Cardiac exam revealed regular rate and rhythm, with an aortic systolic ejection murmur, and pulse oximetry was 94% on both upper limbs and 80% in lower limbs.

**Figure 3:**
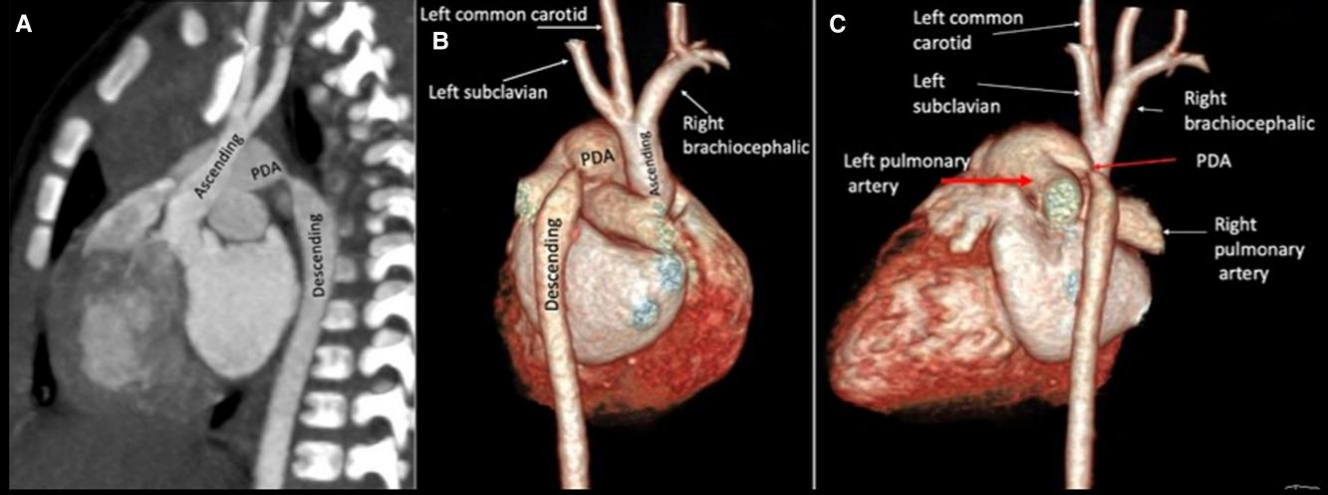
(*A*) Sagittal oblique reformatted image from cardiac computed tomography angiography displays interrupted aortic arch type A. (*B*, *C*) Three-dimensional volume-rendered image shows all three supra-aortic vessels arising from the ascending aorta proximal to the interruption. Patent ductus arteriosus with slight stenosis connects the origin of the left pulmonary artery with the upper descending aorta which lies beyond the interruption.

Preliminary laboratory work did not reveal any abnormalities. An initial echocardiogram showed a tubular structure connecting the left pulmonary artery to the descending aorta. An aortic coarctation was suspected, and a CCTA was performed, displaying an interrupted aortic arch type A and PDA with slight stenosis that connected the origin of the left pulmonary artery with the upper descending aorta, which lies beyond the interruption. (*Figure 3*).

The patient was treated with diuretics and antibiotics, the ligamentum arteriosum was double-ligated, and the interrupted aortic arch was repaired by an anterior patch augmentation technique. Follow-up at 4 weeks showed an optimal clinical evolution.

### Case 4

A five-day-old infant with no prior findings arrived at the emergency department with sudden circulatory shock. He had been discharged from a local clinic after an uncomplicated vaginal delivery.

**Figure 4:**
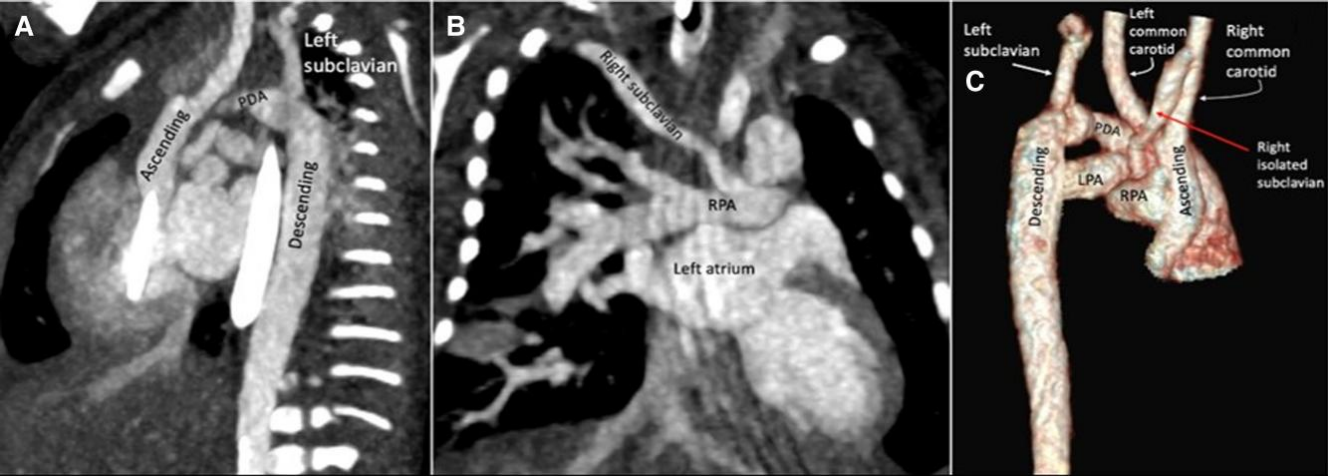
(*A* and *B*) Coronal and lateral oblique reformatted maximum intensity projection images from cardiac computed tomography angiography demonstrate an interrupted aortic arch type B and a right isolated subclavian artery. (*C*) Three-dimensional volume-rendered image in right lateral view shows the interruption occurring beyond the origin of the right and left common carotid. Left subclavian artery arises beyond the interruption. Patent ductus arteriosus continues with the descending aorta. Right isolated subclavian artery originates from the right pulmonary artery.

A TTE was performed, showing a discontinuous aortic arch and a large ventricular septal defect. CCTA demonstrated an interrupted aortic arch type B and a right isolated subclavian artery, left ventricular outflow tract obstruction, pulmonary trunk dilatation, and interventricular septal defect of 10 × 5.6 mm. Three-dimensional volume-rendered image showed the interruption occurring beyond the origin of the right and left common carotid. The left subclavian artery arises beyond the interruption. Patent ductus arteriosus continued with the descending aorta. The right isolated subclavian artery originates from the right pulmonary artery (*Figure 4A–C*).

Immediate haemodynamic stabilization was performed with diuretics, vasoactive drugs, and prostaglandin E1. Surgical correction was performed at one time, with mobilization of the descending aorta and wide anastomosis of the same with the ascending aorta. Unfortunately, the patient died in the post-operative period due to low cardiac output with circulatory shock.

### Case 5

A 50-year-old man presented with a history of resistant hypertension and long-standing claudication. Relevant findings upon initial examination were a mid-systolic murmur in the interscapular area and delayed femoral pulses.

**Figure 5:**
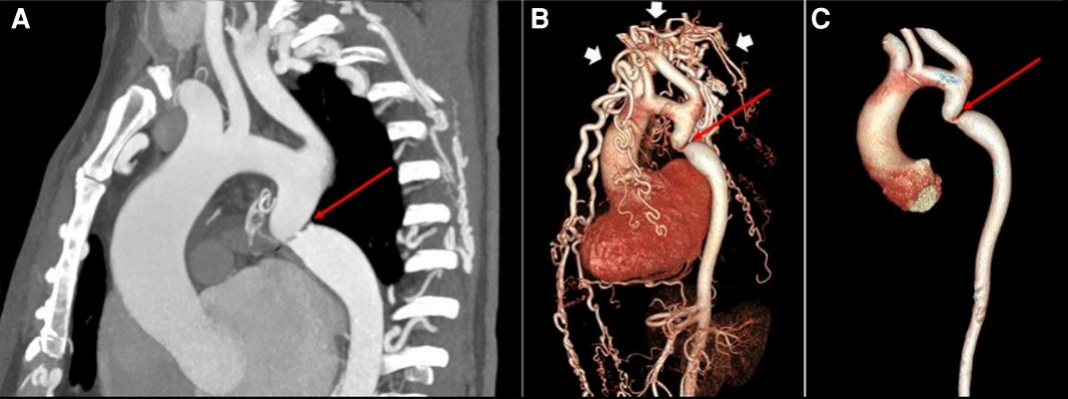
(*A*) Sagittal oblique reformatted maximum intensity projection image from cardiac computed tomography angiography demonstrates a focal severe coarctation (arrow) of the aorta with multiple collateral arteries. (*B* and *C*) Three-dimensional volume-rendered image in left lateral view shows prominent collateral arteries (white arrowheads) due to severe coarctation of the aorta (arrow).

A chest X-ray showed mild cardiomegaly and notching of the inferior aspects of the ribs. A TTE revealed left ventricular hypertrophy and in the suprasternal plane, data compatible with coarctation of the aorta were visualized, so a CCTA was performed, showing a focal severe coarctation in the descending aorta, with multiple collateral arteries. The three-dimensional volume-rendered image in the left lateral view showed prominent internal mammary arteries due to severe coarctation of the aorta (*Figure 5A–C*).

Two overlapping self-expanding stents were implanted, followed by a balloon angioplasty. Follow-up at 6 months was adequate, requiring only metoprolol 100 mg once daily.

### Case 6

A 3-day-old male infant was referred to cardiology due to poor feeding, tachypnoea, and lethargy, and physical examination revealed a systolic murmur. He had been discharged at 24 h after an uncomplicated vaginal delivery.

**Figure 6:**
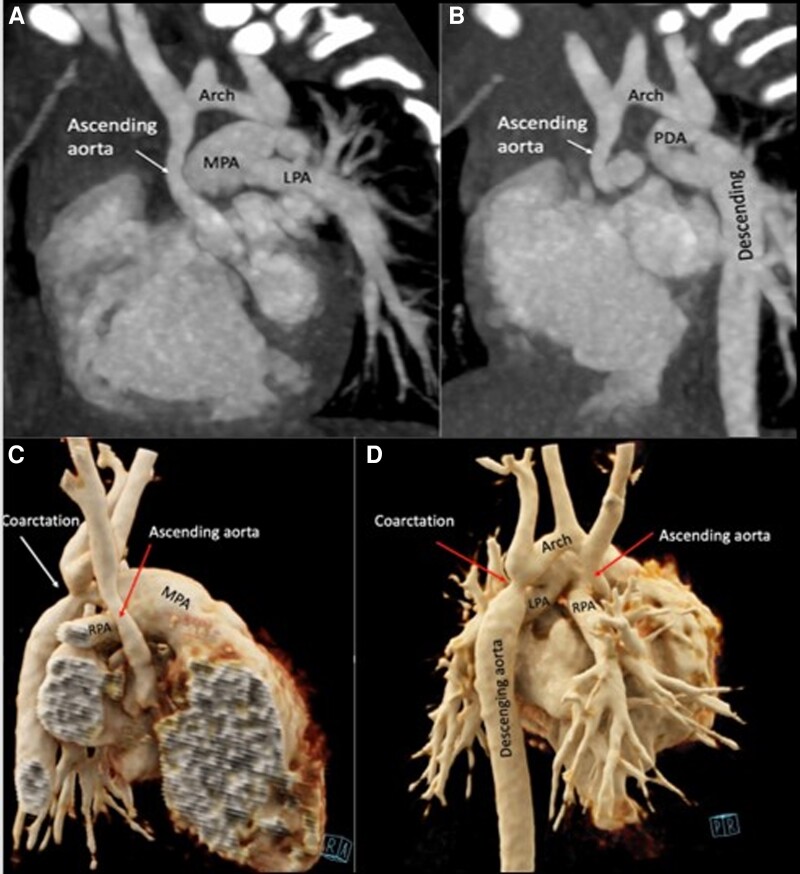
(*A* and *B*) Sagittal oblique reformatted maximum intensity projection image from cardiac computed tomography angiography displays the ascending aorta and aortic arch with pre-ductal coarctation associated with hypoplastic left ventricle syndrome. (*C*, *D*) Three-dimensional volume-rendered image in lateral and posterior views demonstrates diffuse hypoplasia of the aortic root, ascending aorta, and aortic arch.

Diagnosis of hypoplastic left ventricle syndrome was made after the initial TTE that showed dilation of right cavities, mitral atresia, and hypoplasia of left cavities. The CCTA displayed the ascending aorta and aortic arch with pre-ductal coarctation associated with hypoplastic left ventricular syndrome. Three-dimensional CCTA demonstrates diffuse hypoplasia of the aortic root, ascending aorta, and aortic arch (*Figure 6*). Ultra-fast low-dose high pitch CCTA allowed visualization of the aortic arch for surgical planning.

Surgical resection of the coarctation area and end-to-end anastomosis extended to the floor of the aortic arch were performed. Follow-up at 2 months demonstrated a clinically stable patient.

### Case 7

**Figure 7:**
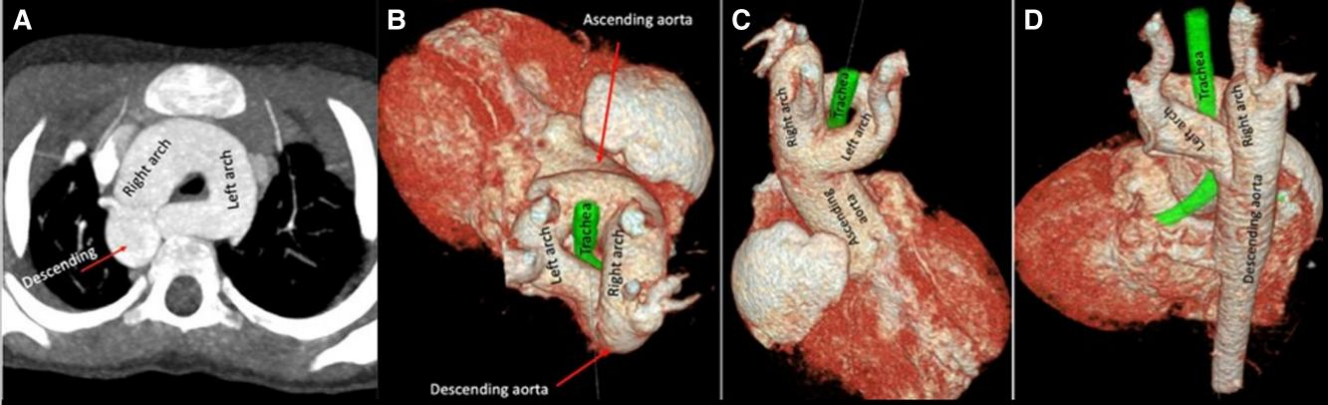
(*A*) Axial oblique reformatted maximum intensity projection images from cardiac computed tomography angiography provide diagnosis of a complete vascular ring made up of a right (dominant) and left aortic arches. This anomaly is the result of persistence of both aortic arches without regression of any segment. (*B*, *C*, *D*) Three-dimensional volume-rendered images in superior, anterior, and posterior views show trachea and oesophagus fully surrounded by two patent aortic arches.

A 9-month-old male presented to the emergency room with acrocyanosis and inadequate weight gain. Physical examination showed inspiratory stridor, and no murmurs were detected. An initial echocardiogram demonstrated pulmonary atresia and interventricular septal defect. CCTA provided the diagnosis of a complete vascular ring made up of a right (dominant) and left aortic arches, with pulmonary atresia, right aortic arch with an aberrant left subclavian artery, and left ligamentum arteriosum. Three-dimensional CCTA showed the trachea and oesophagus fully surrounded by two patent aortic arches (*Figure 7A–D*). Through catheterization, a stent was placed in the left aortic arch. By medical-surgical consensus, it was decided that the patient is not a candidate for a corrective procedure at this time, mainly because he remained clinically and haemodynamically stable. At the three months follow-up, the patient remains in stable condition.

Supplementary material online, *table S1*.

## Discussion

CAoD comprise an uncommon spectrum of vascular anomalies, which includes obstructions in the aortic arch (coarctation, hypoplasia, and interruption) and vascular rings. Echocardiography, cardiac magnetic resonance imaging, and computed tomography angiography are important imaging modalities used to identify and diagnose aortic arch variants and anomalies.^4^

We present seven case reports of CAoD in which the clinical manifestations throughout the cases are discussed, highlighting the heterogeneity of the symptoms and the importance of using high-resolution imaging modalities.

Adequate assessment allows early diagnosis of the aortic diseases,^5^ which are often inversely proportional to their severity.

Multi-imaging techniques are indispensable for the assessment of CAoD, as they demonstrate the complex aortic arch anomalies and provide an adequate evaluation of the surrounding structures, such as the heart, trachea, and oesophagus.

CCTA three-dimensional reconstructions provide detailed images of vascular rings and Kommerell diverticulum and avoid more complex procedures, such as bronchoscopy and barium oesophagogram.

CCTA has superior accuracy to visualize the coronary arteries as well as the great vessels, and this technique is essential in pre-procedural planning of the aortic arch pathology.

Multislice computed tomography angiography provides detailed information of the structure of the vessel; it is outstanding in displaying the distal aortic arch, brachiocephalic artery, and the spatial relationship between the trachea, bronchi, and aortic arch. Three-dimensional-static images and rotated reconstruction images can be generated by CT, which provide specific information of a certain site and can be rotated from every direction for analysing pre- and post-operative anatomy changes.^6^

Other advantages of CCTA are its ability to determine the coronary artery dominance, angulation of the aortic root, ostial narrowing, presence, and length of the intramural course, which cannot be reliably determined by echocardiography. Even though echocardiography is a fundamental tool for the assessment of cardiac pathologies, it is somehow limited for visualizing extracardiac structures such as certain aortic segments and is operator dependent, and an optimal acoustic window may be difficult to obtain in older children or adults. On the other hand, MRI is useful for evaluating congenital coronary anomalies in older children and adolescents but is markedly limited in the youngest patients due to image quality being related to both patient age and heart rate. Other downsides are the higher cost, limited availability, and image degrading artefacts due to implanted stents and coils.^7,8^

The faster image acquisition time and excellent images of the great vessels obtained by the CCTA allow it to be compared to cardiac MRI, but the two methods should be seen as complementary to each other.

Radiation exposure remains the main limitation for CCTA compared to MRI, but shorter acquisition times make CCTA a more suitable option for the assessment of pediatric and claustrophobic patients, in which adequate immobilization may be hard to achieve and requires the potential use of anaesthesia.

It is essential to highlight that new-generation CT scanners provide an important radiation dose reduction, as shown in the study of Timotheus *et al*. where the median dose length product for patients in the CT group was 9 mGy ∗ cm (range 5–493). For all ages, the median effective doses for the CT group were 0.74 mSv (range 0.43–15.31).^9^

In the study of Walsh *et al*., mean number of CT scans performed was 0.44 ± 0.4 (0–11), and the mean exposure was 352 μGym^2^, giving a mean cumulative total of 154 μGym^2^ (range 0–3872) per person.^10^ In our study, the median effective dose was 0.67 mSv (see supplementary material online, *table S2*).

Three-dimensional volume-rendered images of arterial anatomy are a valuable tool for optimal diagnosis and surgical planning, as observed in many of our cases.

Three-dimensional printing has been shown to have a positive impact on the pre-operative planning of corrective surgery, due to the excellent geometric information that it provides.^6,10,11^

Familiarity with the spectrum of anomalies encompassed in the aortic arch is essential for a timely diagnosis, and the basic knowledge of the different imaging modalities used in these pathologies is extremely important, as they significantly guide the management, which includes surgical and percutaneous interventions.

## Supplementary Material

ytad155_Supplementary_Data

## Data Availability

The data underlying this article will be shared on reasonable request to the corresponding author.

## References

[1] Landeras LA , Chung JH. Congenital thoracic aortic disease. Radiol Clin North Am 2019;57:113–125. 30454807 10.1016/j.rcl.2018.08.008

[2] Russo V , Renzulli M, La Palombara C, Fattori R. Congenital diseases of the thoracic aorta. Role of MRI and MRA. Eur Radiol 2006;16:676–684. 16249863 10.1007/s00330-005-0027-y

[3] Türkvatan A , Büyükbayraktar FG, Ölçer T, Cumhur T. Congenital anomalies of the aortic arch: evaluation with the use of multidetector computed tomography. Korean J Radiol 2009;10:176. 19270864 10.3348/kjr.2009.10.2.176 PMC2651449

[4] Hanneman K , Newman B, Chan F. Congenital variants and anomalies of the aortic arch. RadioGraphics 2017;37:32–51. 27860551 10.1148/rg.2017160033

[5] Yoshimura N , Fukahara K, Yamashita A, Doi T, Yamashita S, Homma T, et al Congenital vascular ring. Surg Today 2020;50:1151–1158. 31676999 10.1007/s00595-019-01907-5

[6] Zhu X , Wu C, He Y, Qin B, Yang H, Huang H, et al 3D-imaging evaluation of double aortic arch with MSCTA: a case report and mini-review. J X-Ray Sci Technol 2018;26:103–109. 10.3233/XST-17270 28854527

[7] Han BK , Rigsby CK, Hlavacek A, Leipsic J, Nicol ED, Siegel MJ, et al Computed tomography imaging in patients with congenital heart disease part I: rationale and utility. An expert consensus document of the Society of Cardiovascular Computed Tomography (SCCT). J Cardiovasc Comput Tomogr 2015;9:475–492. 26272851 10.1016/j.jcct.2015.07.004

[8] Sachdeva S , Gupta SK. Imaging modalities in congenital heart disease. Indian J Pediatr 2020;87:385–397. 32285327 10.1007/s12098-020-03209-y

[9] Watson TG , Mah E, Joseph Schoepf U, King L, Huda W, Hlavacek AM. Effective radiation dose in computed tomographic angiography of the chest and diagnostic cardiac catheterization in pediatric patients. Pediatr Cardiol 2013;34:518–524. 22956060 10.1007/s00246-012-0486-2

[10] Walsh MA , Noga M, Rutledge J. Cumulative radiation exposure in pediatric patients with congenital heart disease. Pediatr Cardiol 2015;36:289–294. 25124721 10.1007/s00246-014-0999-y

[11] Lau I , Wong YH, Yeong CH, Abdul Aziz YF, Md Sari NA, Hashim SA, et al Quantitative and qualitative comparison of low- and high-cost 3D-printed heart models. Quant Imaging Med Surg 2019;9:107–114. 30788252 10.21037/qims.2019.01.02 PMC6351814

