# Supplementary material for: Cardiac computed tomography assessment of congenital aortic diseases: a case series
Source: Eur Heart J Case Rep. 2023 Mar 30;7(4):ytad155. doi: 10.1093/ehjcr/ytad155 (PMC10108973; doi:10.1093/ehjcr/ytad155)
Supplement: ytad155_Supplementary_Data [file ytad155_supplementary_data.zip › Suppl table 1.docx]

Supplementary table 1. Differences between Cardiac Computed Tomography and Echocardiography in each case.

|  | Case 1 | Case 2 | Case 3 | Case 4 | Case 5 | Case 6 | Case 7 |
| --- | --- | --- | --- | --- | --- | --- | --- |
| Cardiac Computed Tomography | Complete vascular ring, left Kommerell diverticulum, CIV, and PDA | Complete loose vascular ring, right Kommerell diverticulum, and a right ligamentum arteriosum | Interrupted aortic arch type A and PDA with slight stenosis | Interrupted aortic arch type B and a right isolated subclavian artery | Focal severe coarctation in descending aorta, with multiple collateral arteries | Displayed the ascending aorta and aortic arch with preductal coarctation, hypoplastic left ventricle syndrome. Hypoplasia of the aortic root, ascending aorta, and aortic arch | Complete vascular ring, pulmonary atresia, trachea, and esophagus fully surrounded |
| Echocardiography | Right-sided aortic arch | No evidence of cardiac malformations | Connection between the left pulmonary artery to the descending aorta | Discontinuous aortic arch and a large ventricular septal defect | Coarctation of the aorta | Mitral atresia, dilation of right cavities, and hypoplasia of left cavities | Pulmonary atresia and interventricular communication |
