# Supplementary material for: Cardiac computed tomography assessment of congenital aortic diseases: a case series
Source: Eur Heart J Case Rep. 2023 Mar 30;7(4):ytad155. doi: 10.1093/ehjcr/ytad155 (PMC10108973; doi:10.1093/ehjcr/ytad155)
Supplement: ytad155_Supplementary_Data [file ytad155_supplementary_data.zip › Suppl table 2.docx]

Supplementary table 2. Radiation exposure in infants. Comparison between two concentration centers, and the NICICh.

|  | Case 1 | | Case 2 | | | Case 3 | | Case 4 | | Case 5 | | Case 6 | | Case 7 | |
| --- | --- | --- | --- | --- | --- | --- | --- | --- | --- | --- | --- | --- | --- | --- | --- |
| Radiation exposure | DLP mGcm | MDE msv | DLP mGcm | | MDE msv | DLP mGcm | MDE msv | DLP mGcm | MDE msv | DLP mGcm | MDE msv | DLP mGcm | MDE msv | DLP mGcm | MDE msv |
| NICICh | 38 | .494 | 31 | | .558 | 31.1 | .4043 | 34 | .612 | 42 | 1.63 | 16 | .288 | 42 | 0.75 |
| Timotheus et.al. | | | | | | | | Walsh et. al. | | | | | | | |
| DLP: 9 mGy*cm (5-493) | | | | MDE: 0.74 (0.43-15.31) msv | | | | DLP: 352 µGym2 | | | | Mean cumulative total: 154 µGym2 (range 0–3,872) | | | |

Abbreviations: NICICh: National Institute of Cardiology Ignacio Chavez; DPL dose length product; MDE median effective doses
