# Supplementary material for: Cardiac computed tomography assessment of congenital aortic diseases: a case series
Source: Eur Heart J Case Rep. 2023 Mar 30;7(4):ytad155. doi: 10.1093/ehjcr/ytad155 (PMC10108973; doi:10.1093/ehjcr/ytad155)
Supplement: ytad155_Supplementary_Data [file ytad155_supplementary_data.zip › EHJ-CR-Slide-Set_V2.pptx]

## Slide 1
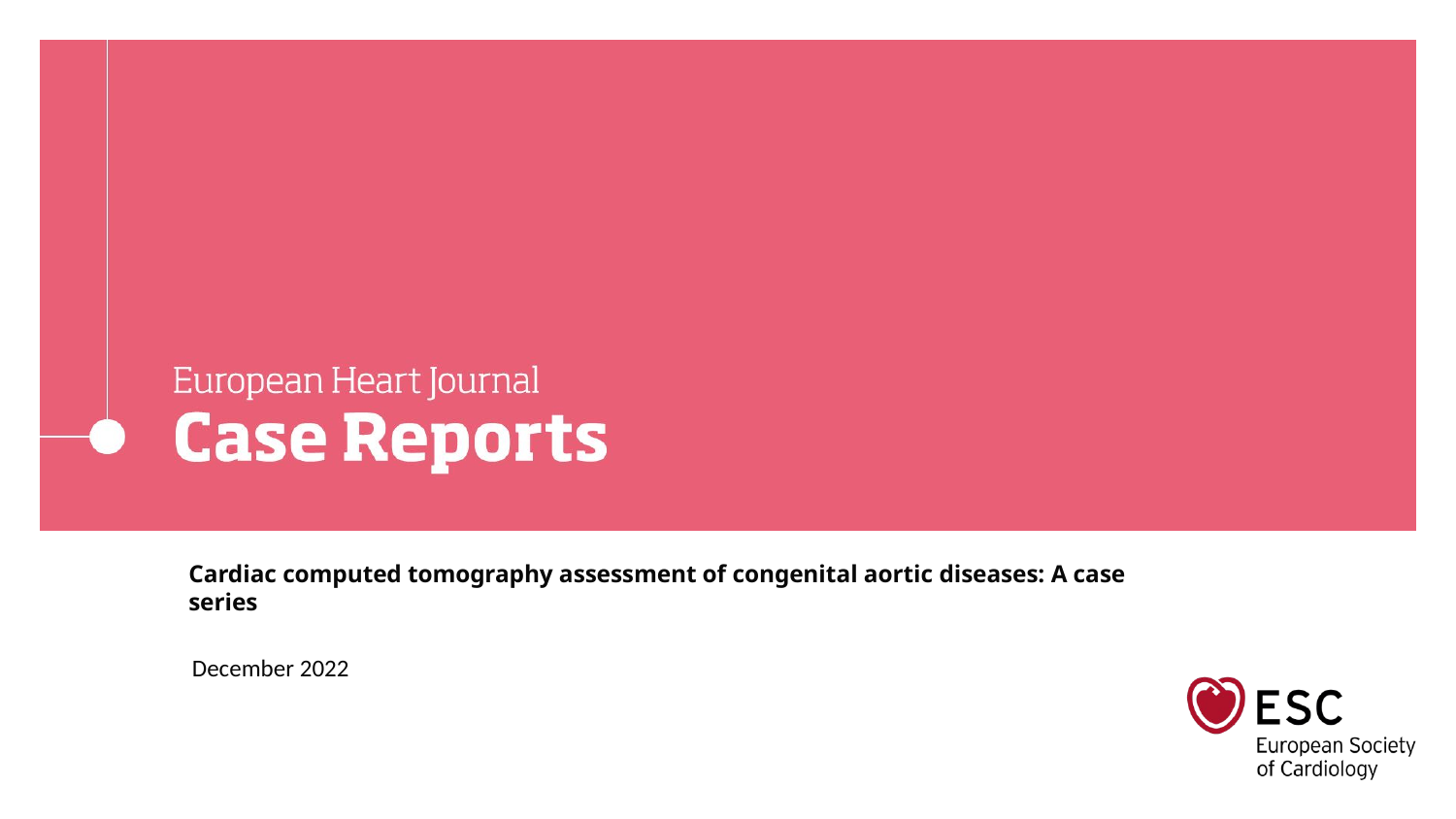

# Cardiac computed tomography assessment of congenital aortic diseases: A case series
December 2022

## Slide 2
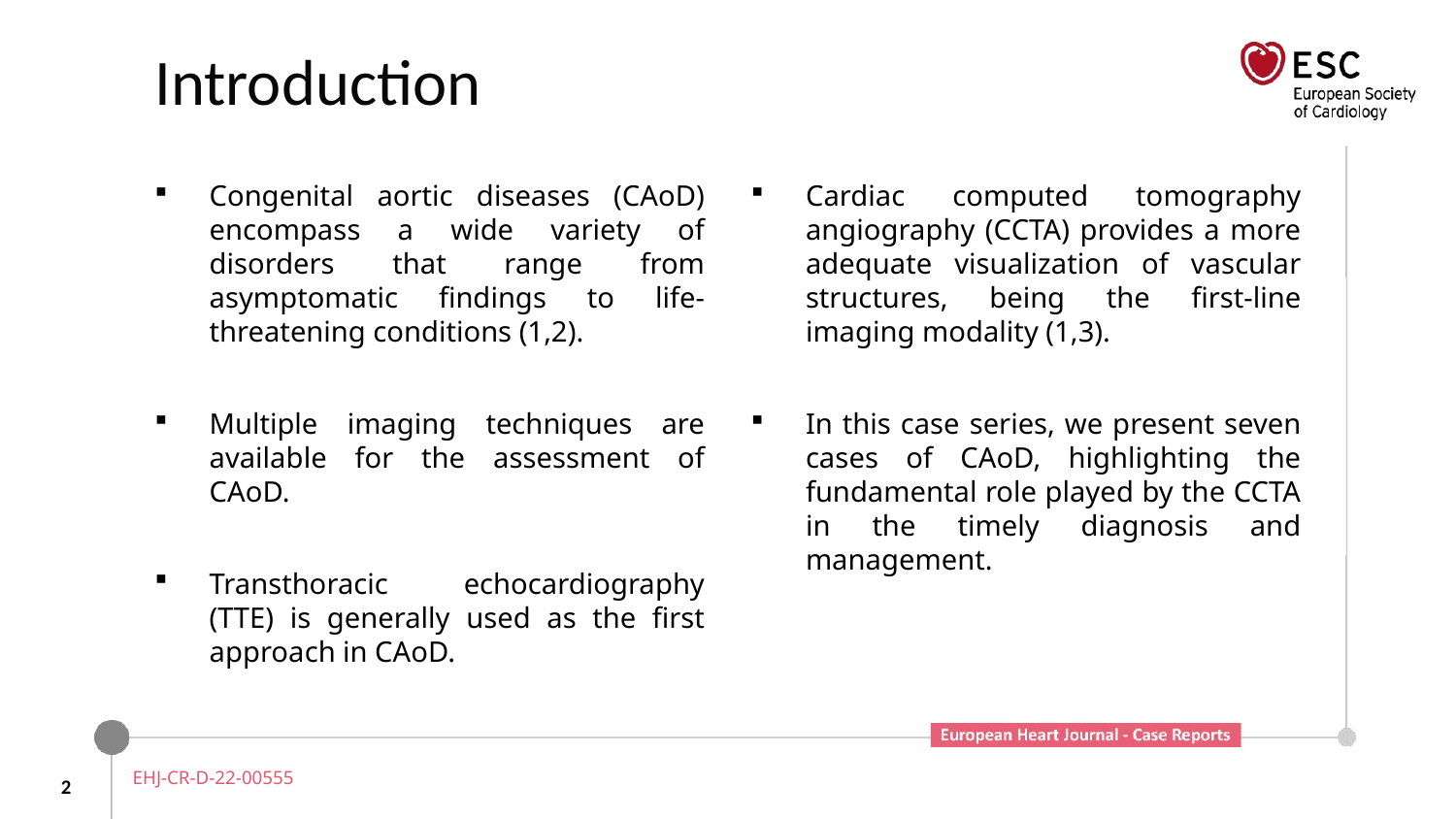

# Introduction
Congenital aortic diseases (CAoD) encompass a wide variety of disorders that range from asymptomatic findings to life-threatening conditions (1,2).
Multiple imaging techniques are available for the assessment of CAoD.
Transthoracic echocardiography (TTE) is generally used as the first approach in CAoD.
Cardiac computed tomography angiography (CCTA) provides a more adequate visualization of vascular structures, being the first-line imaging modality (1,3).
In this case series, we present seven cases of CAoD, highlighting the fundamental role played by the CCTA in the timely diagnosis and management.
EHJ-CR-D-22-00555
2

## Slide 3
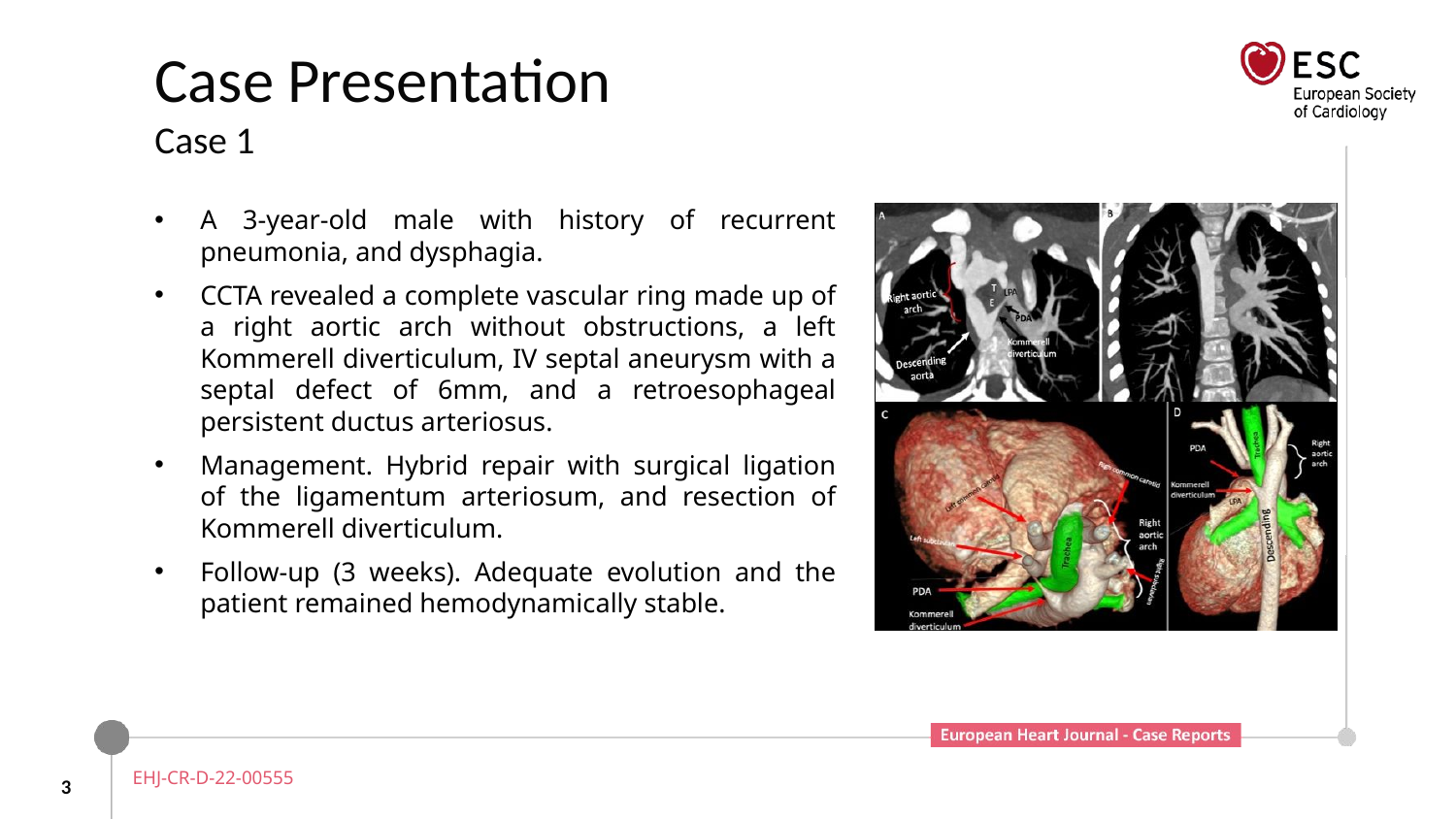

# Case Presentation Case 1
A 3-year-old male with history of recurrent pneumonia, and dysphagia.
CCTA revealed a complete vascular ring made up of a right aortic arch without obstructions, a left Kommerell diverticulum, IV septal aneurysm with a septal defect of 6mm, and a retroesophageal persistent ductus arteriosus.
Management. Hybrid repair with surgical ligation of the ligamentum arteriosum, and resection of Kommerell diverticulum.
Follow-up (3 weeks). Adequate evolution and the patient remained hemodynamically stable.
EHJ-CR-D-22-00555
3

## Slide 4
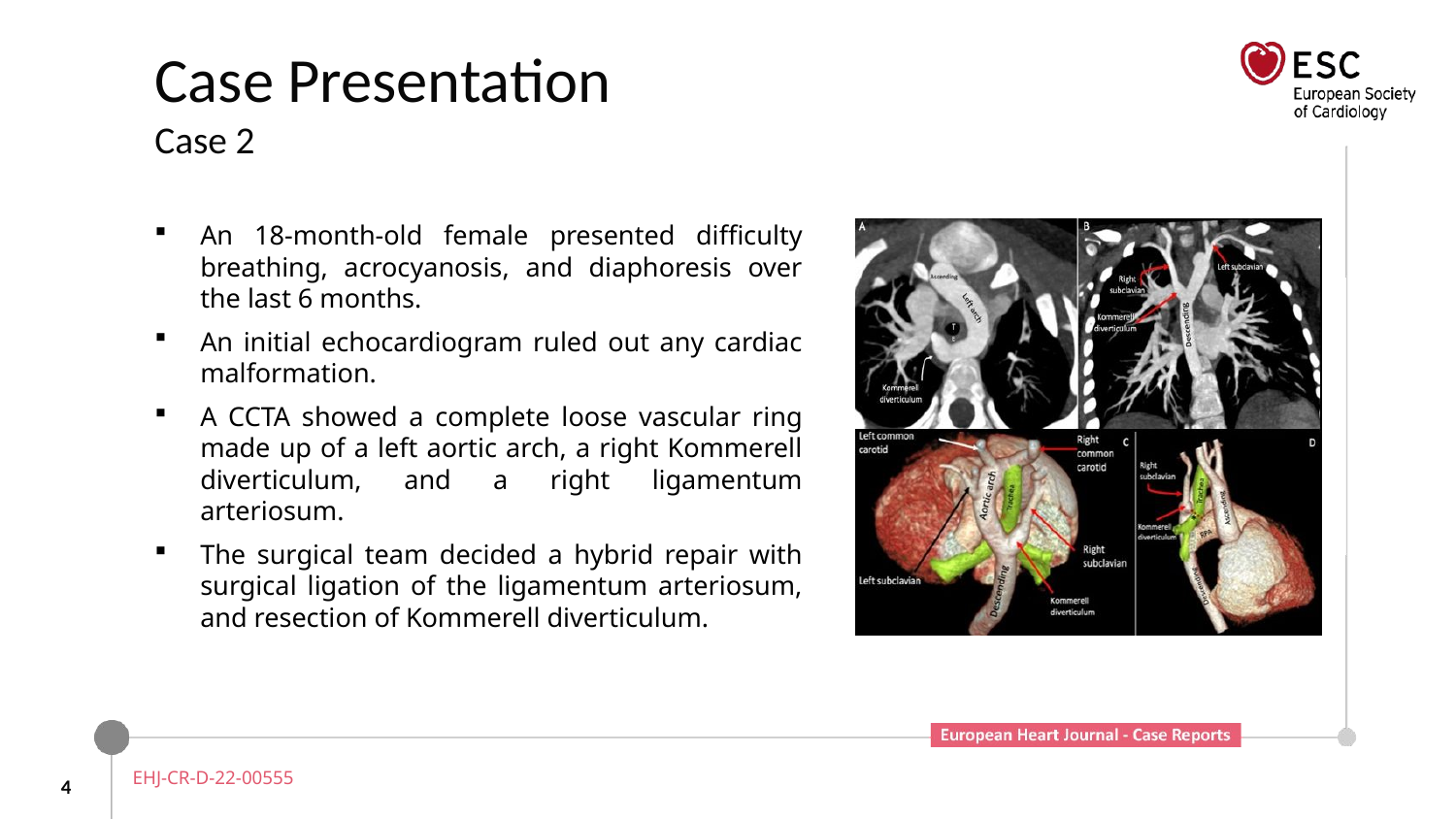

# Case Presentation Case 2
An 18-month-old female presented difficulty breathing, acrocyanosis, and diaphoresis over the last 6 months.
An initial echocardiogram ruled out any cardiac malformation.
A CCTA showed a complete loose vascular ring made up of a left aortic arch, a right Kommerell diverticulum, and a right ligamentum arteriosum.
The surgical team decided a hybrid repair with surgical ligation of the ligamentum arteriosum, and resection of Kommerell diverticulum.
EHJ-CR-D-22-00555
4

## Slide 5
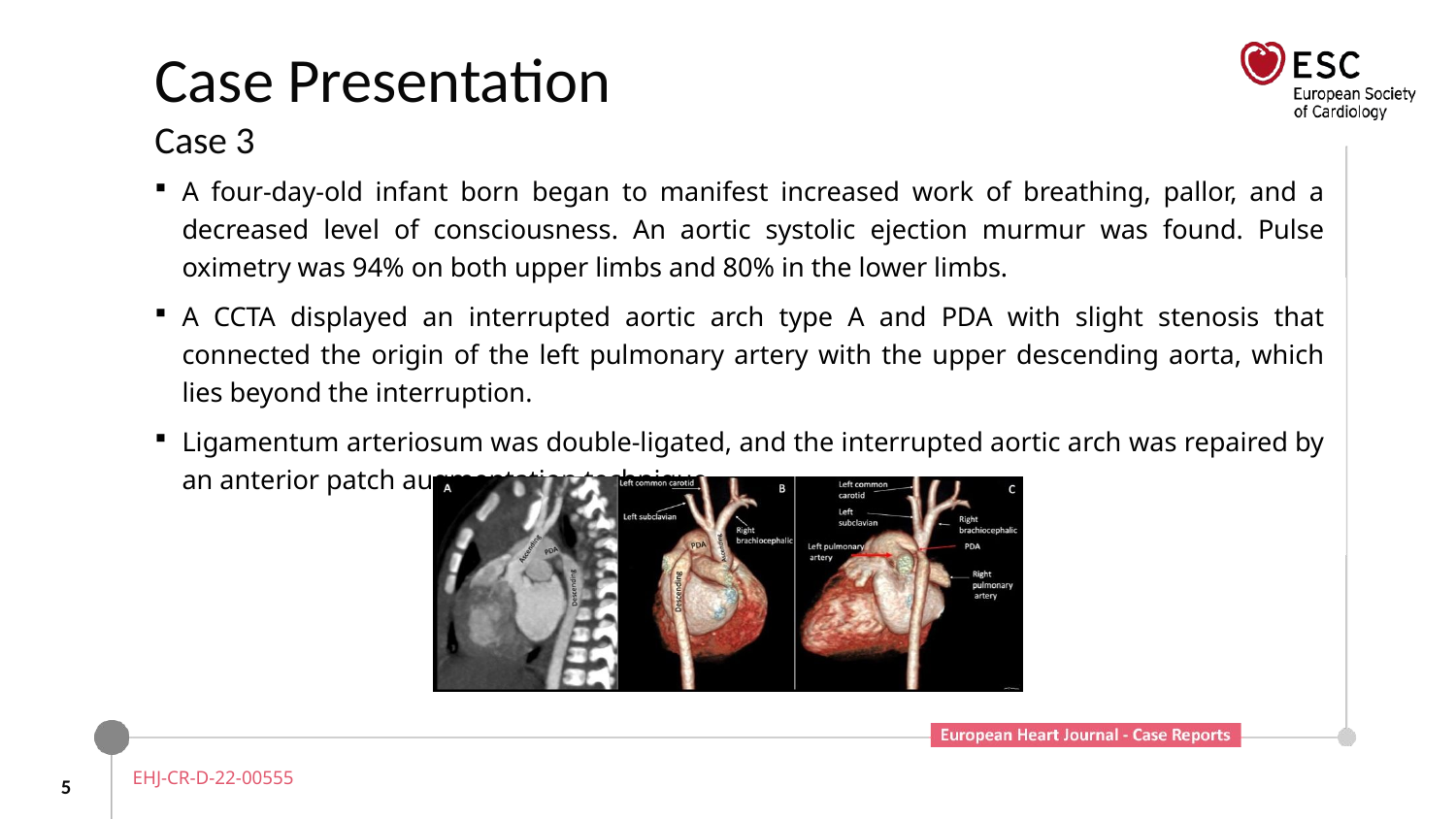

# Case Presentation Case 3
A four-day-old infant born began to manifest increased work of breathing, pallor, and a decreased level of consciousness. An aortic systolic ejection murmur was found. Pulse oximetry was 94% on both upper limbs and 80% in the lower limbs.
A CCTA displayed an interrupted aortic arch type A and PDA with slight stenosis that connected the origin of the left pulmonary artery with the upper descending aorta, which lies beyond the interruption.
Ligamentum arteriosum was double-ligated, and the interrupted aortic arch was repaired by an anterior patch augmentation technique.
EHJ-CR-D-22-00555
5

## Slide 6
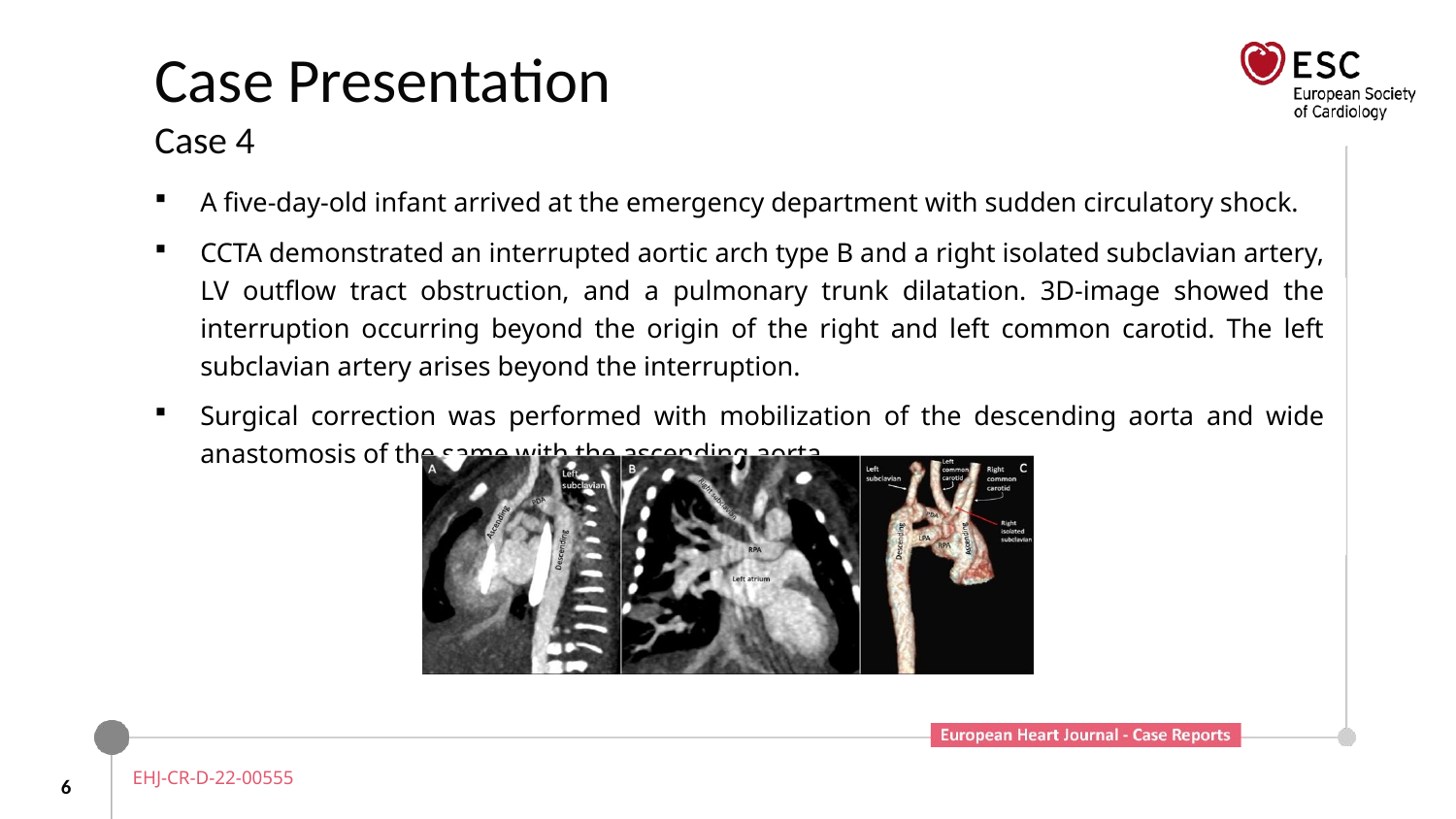

# Case Presentation Case 4
A five-day-old infant arrived at the emergency department with sudden circulatory shock.
CCTA demonstrated an interrupted aortic arch type B and a right isolated subclavian artery, LV outflow tract obstruction, and a pulmonary trunk dilatation. 3D-image showed the interruption occurring beyond the origin of the right and left common carotid. The left subclavian artery arises beyond the interruption.
Surgical correction was performed with mobilization of the descending aorta and wide anastomosis of the same with the ascending aorta.
EHJ-CR-D-22-00555
6

## Slide 7
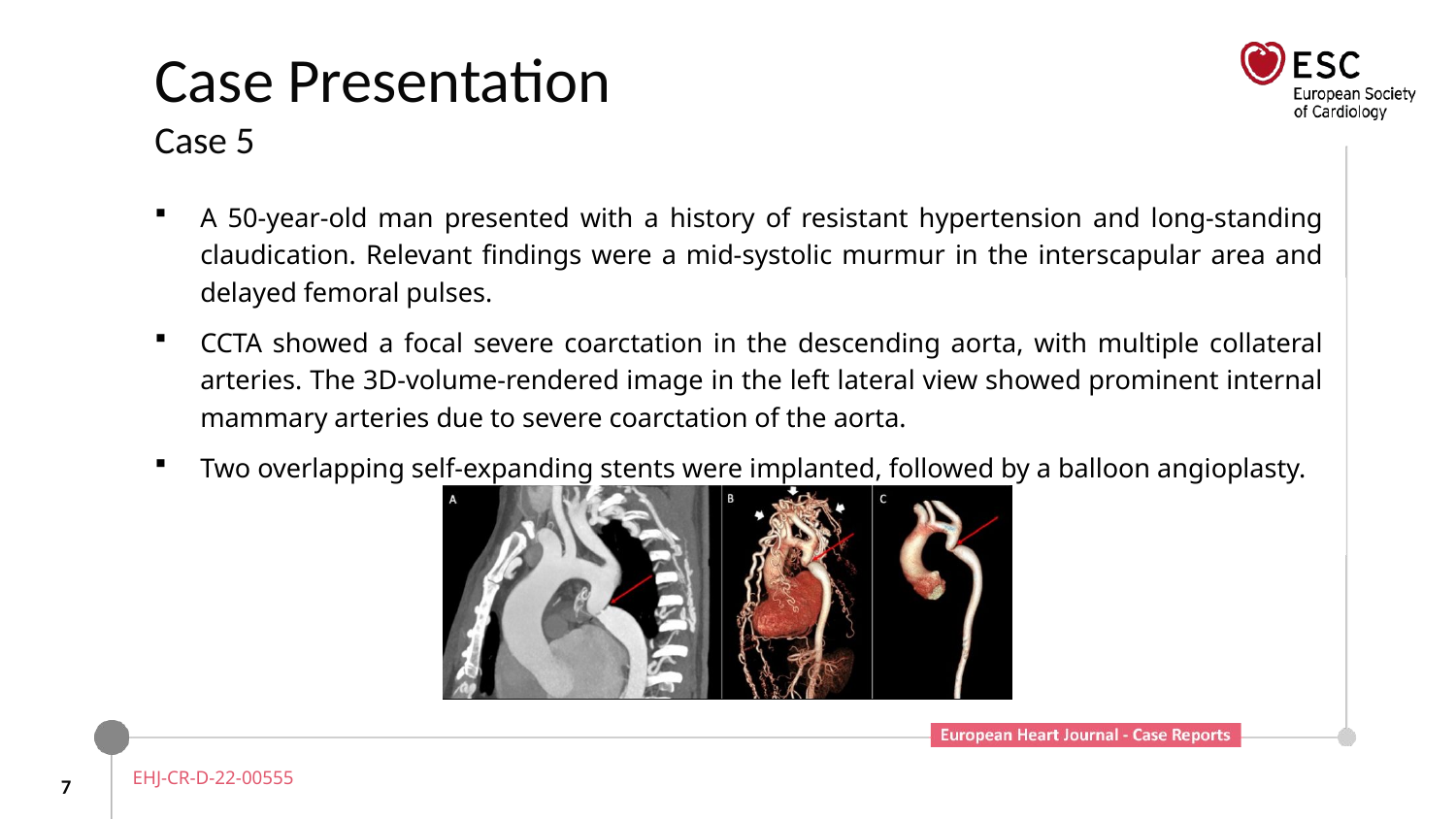

# Case Presentation Case 5
A 50-year-old man presented with a history of resistant hypertension and long-standing claudication. Relevant findings were a mid-systolic murmur in the interscapular area and delayed femoral pulses.
CCTA showed a focal severe coarctation in the descending aorta, with multiple collateral arteries. The 3D-volume-rendered image in the left lateral view showed prominent internal mammary arteries due to severe coarctation of the aorta.
Two overlapping self-expanding stents were implanted, followed by a balloon angioplasty.
EHJ-CR-D-22-00555
7

## Slide 8
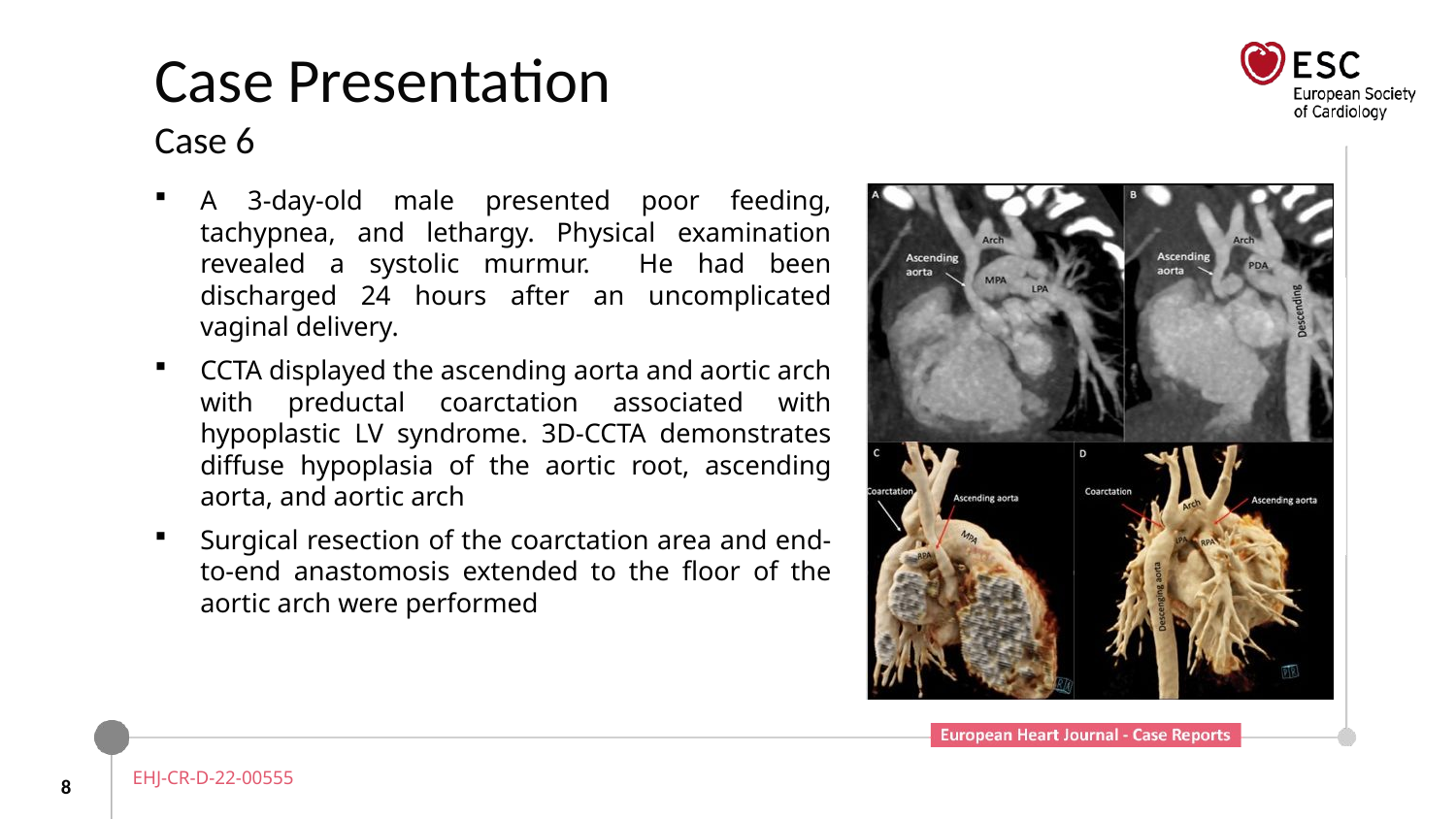

# Case Presentation Case 6
A 3-day-old male presented poor feeding, tachypnea, and lethargy. Physical examination revealed a systolic murmur. He had been discharged 24 hours after an uncomplicated vaginal delivery.
CCTA displayed the ascending aorta and aortic arch with preductal coarctation associated with hypoplastic LV syndrome. 3D-CCTA demonstrates diffuse hypoplasia of the aortic root, ascending aorta, and aortic arch
Surgical resection of the coarctation area and end-to-end anastomosis extended to the floor of the aortic arch were performed
EHJ-CR-D-22-00555
8

## Slide 9
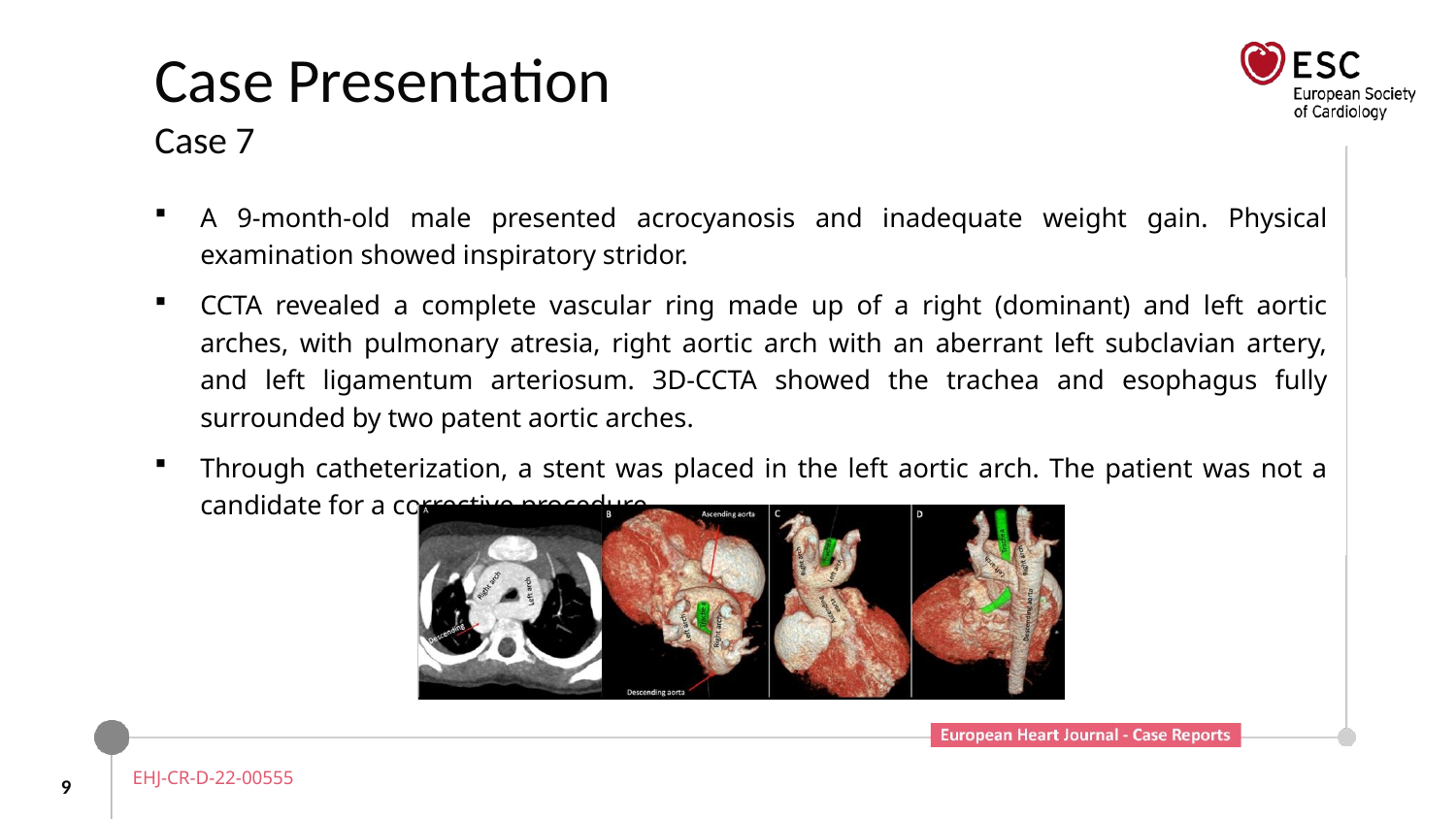

# Case Presentation Case 7
A 9-month-old male presented acrocyanosis and inadequate weight gain. Physical examination showed inspiratory stridor.
CCTA revealed a complete vascular ring made up of a right (dominant) and left aortic arches, with pulmonary atresia, right aortic arch with an aberrant left subclavian artery, and left ligamentum arteriosum. 3D-CCTA showed the trachea and esophagus fully surrounded by two patent aortic arches.
Through catheterization, a stent was placed in the left aortic arch. The patient was not a candidate for a corrective procedure.
EHJ-CR-D-22-00555
9

## Slide 10
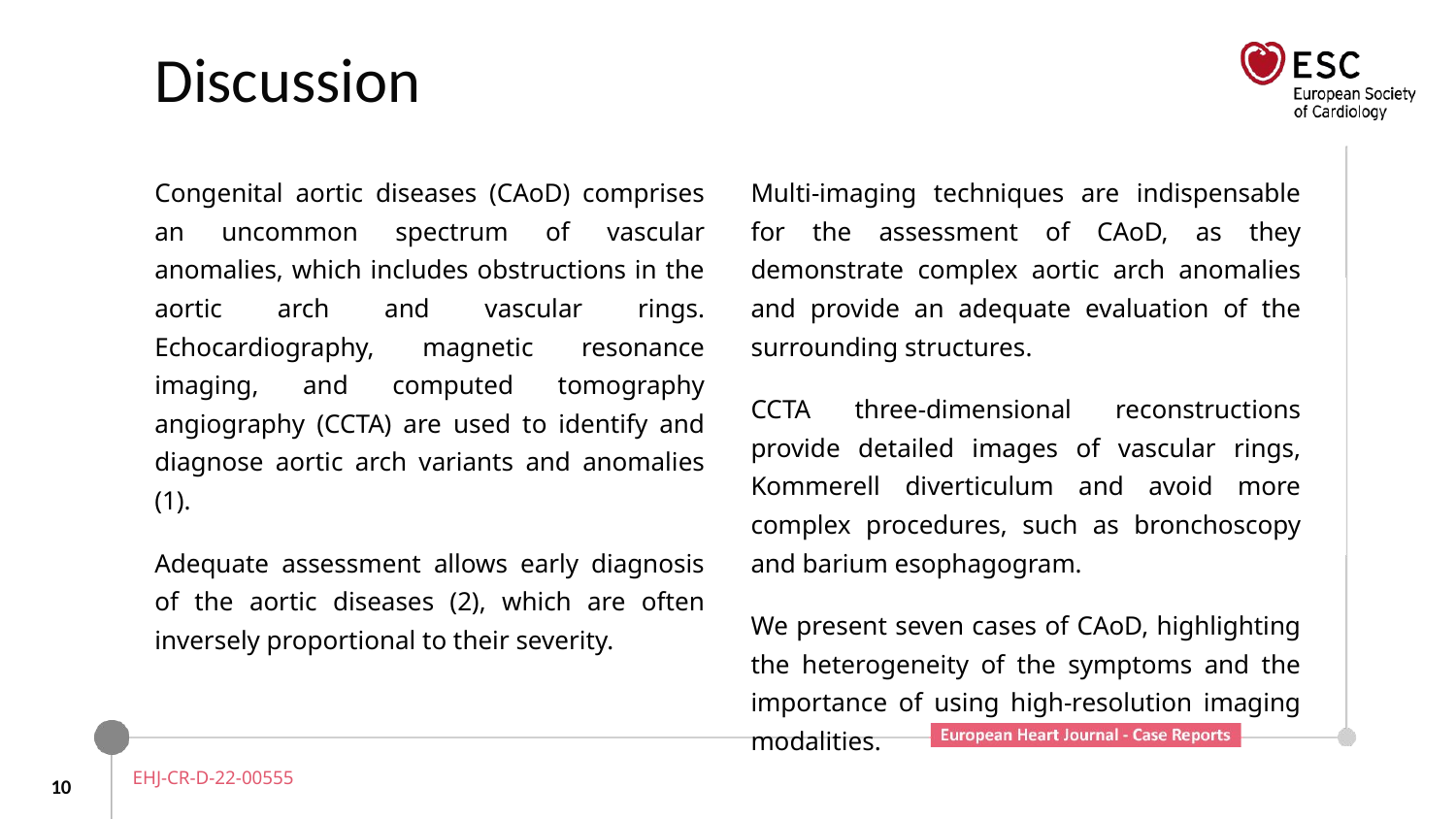

# Discussion
Congenital aortic diseases (CAoD) comprises an uncommon spectrum of vascular anomalies, which includes obstructions in the aortic arch and vascular rings. Echocardiography, magnetic resonance imaging, and computed tomography angiography (CCTA) are used to identify and diagnose aortic arch variants and anomalies (1).
Adequate assessment allows early diagnosis of the aortic diseases (2), which are often inversely proportional to their severity.
Multi-imaging techniques are indispensable for the assessment of CAoD, as they demonstrate complex aortic arch anomalies and provide an adequate evaluation of the surrounding structures.
CCTA three-dimensional reconstructions provide detailed images of vascular rings, Kommerell diverticulum and avoid more complex procedures, such as bronchoscopy and barium esophagogram.
We present seven cases of CAoD, highlighting the heterogeneity of the symptoms and the importance of using high-resolution imaging modalities.
EHJ-CR-D-22-00555
10

## Slide 11
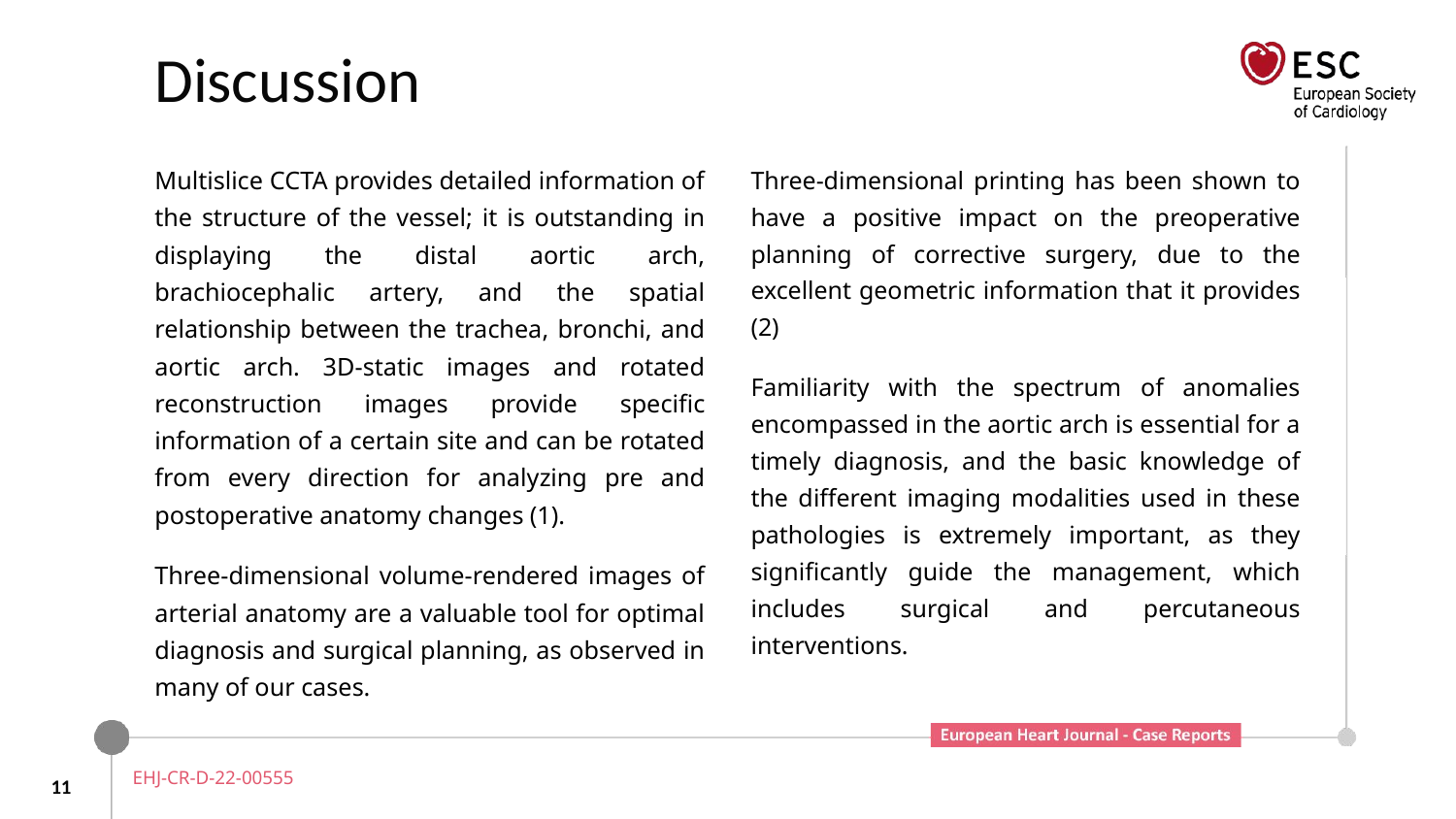

# Discussion
Multislice CCTA provides detailed information of the structure of the vessel; it is outstanding in displaying the distal aortic arch, brachiocephalic artery, and the spatial relationship between the trachea, bronchi, and aortic arch. 3D-static images and rotated reconstruction images provide specific information of a certain site and can be rotated from every direction for analyzing pre and postoperative anatomy changes (1).
Three-dimensional volume-rendered images of arterial anatomy are a valuable tool for optimal diagnosis and surgical planning, as observed in many of our cases.
Three-dimensional printing has been shown to have a positive impact on the preoperative planning of corrective surgery, due to the excellent geometric information that it provides (2)
Familiarity with the spectrum of anomalies encompassed in the aortic arch is essential for a timely diagnosis, and the basic knowledge of the different imaging modalities used in these pathologies is extremely important, as they significantly guide the management, which includes surgical and percutaneous interventions.
EHJ-CR-D-22-00555
11

## Slide 12
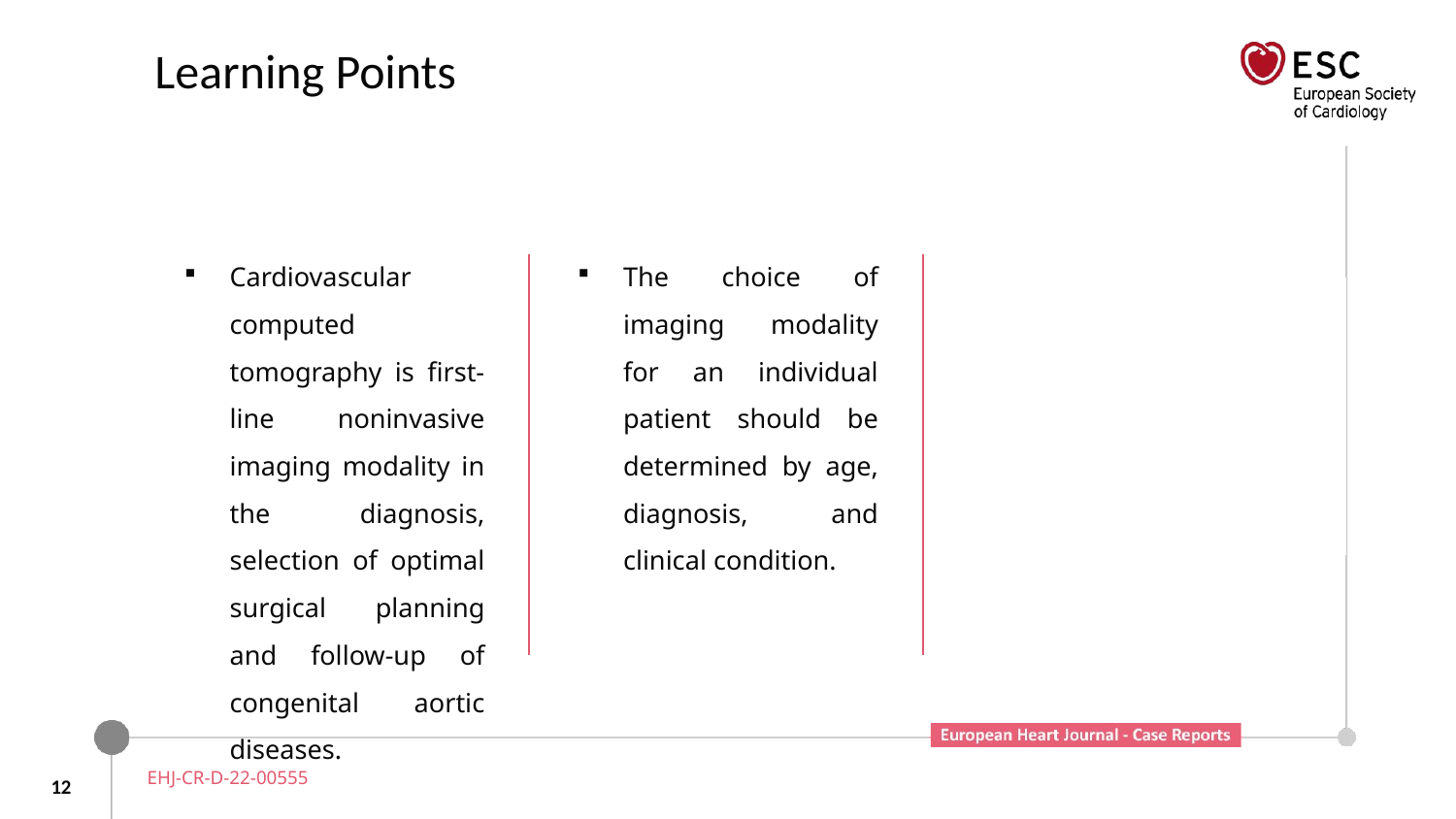

# Learning Points
Cardiovascular computed tomography is first-line noninvasive imaging modality in the diagnosis, selection of optimal surgical planning and follow-up of congenital aortic diseases.
The choice of imaging modality for an individual patient should be determined by age, diagnosis, and clinical condition.
EHJ-CR-D-22-00555
12

## Slide 13
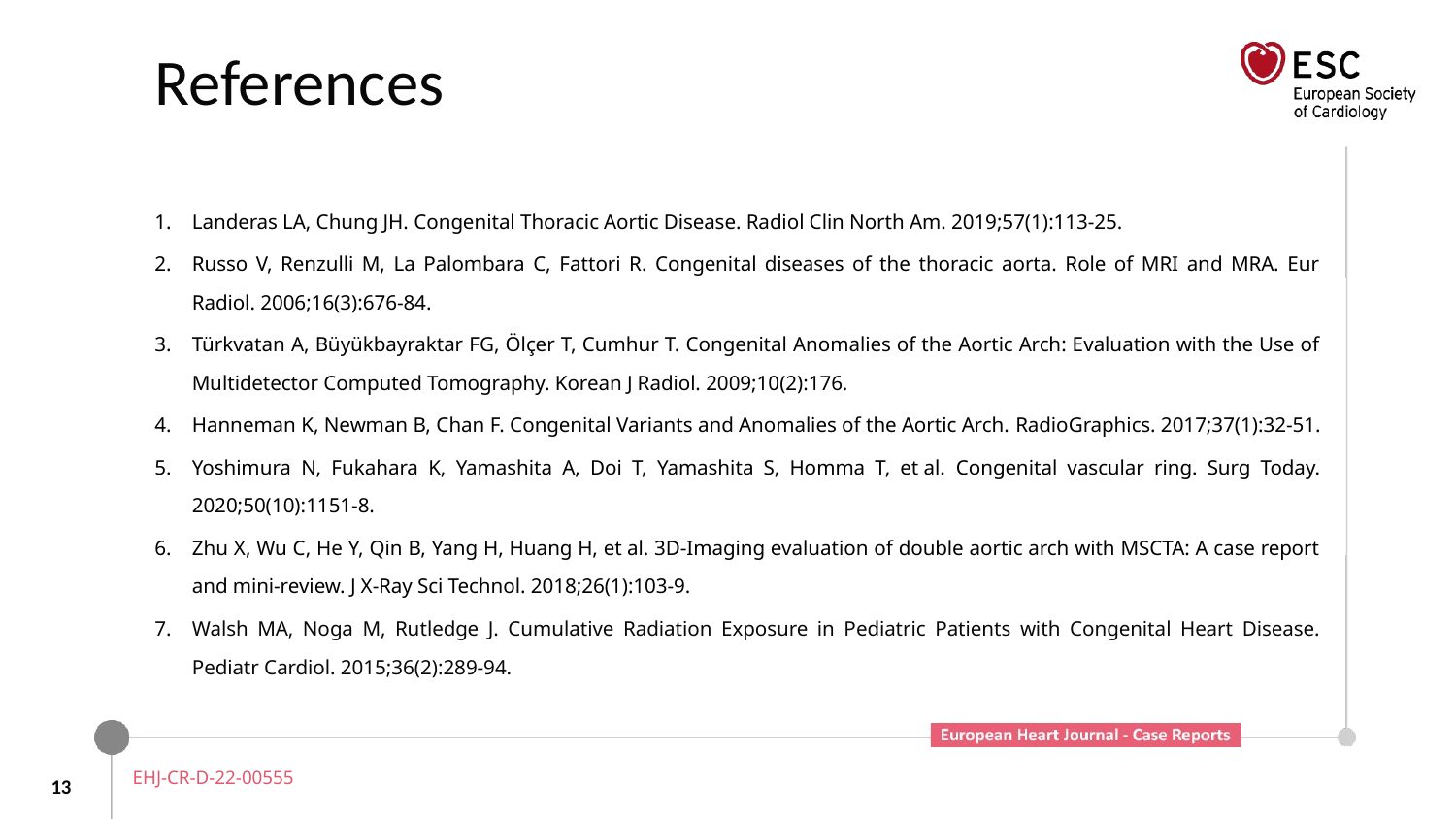

# References
Landeras LA, Chung JH. Congenital Thoracic Aortic Disease. Radiol Clin North Am. 2019;57(1):113-25.
Russo V, Renzulli M, La Palombara C, Fattori R. Congenital diseases of the thoracic aorta. Role of MRI and MRA. Eur Radiol. 2006;16(3):676-84.
Türkvatan A, Büyükbayraktar FG, Ölçer T, Cumhur T. Congenital Anomalies of the Aortic Arch: Evaluation with the Use of Multidetector Computed Tomography. Korean J Radiol. 2009;10(2):176.
Hanneman K, Newman B, Chan F. Congenital Variants and Anomalies of the Aortic Arch. RadioGraphics. 2017;37(1):32-51.
Yoshimura N, Fukahara K, Yamashita A, Doi T, Yamashita S, Homma T, et al. Congenital vascular ring. Surg Today. 2020;50(10):1151-8.
Zhu X, Wu C, He Y, Qin B, Yang H, Huang H, et al. 3D-Imaging evaluation of double aortic arch with MSCTA: A case report and mini-review. J X-Ray Sci Technol. 2018;26(1):103-9.
Walsh MA, Noga M, Rutledge J. Cumulative Radiation Exposure in Pediatric Patients with Congenital Heart Disease. Pediatr Cardiol. 2015;36(2):289-94.
EHJ-CR-D-22-00555
13
